# UPLC-MS-ESI-QTOF Analysis and Antifungal Activity of Aqueous Extracts of *Spondias tuberosa*

**DOI:** 10.3390/molecules28010305

**Published:** 2022-12-30

**Authors:** Antonia Thassya Lucas dos Santos, Joara Nályda Pereira Carneiro, Rafael Pereira da Cruz, Débora Lima Sales, Jacqueline Cosmo Andrade-Pinheiro, Maria Audilene de Freitas, Marta Regina Kerntopf, Gyllyandeson de Araújo Delmondes, Paulo Riceli Vasconcelos Ribeiro, Edy Sousa de Brito, Francisco Lucas Alves Batista, Francisco Ernani Alves Magalhães, Ivo C. Pita Neto, Maria Flaviana Bezerra Morais-Braga, Radosław Kowalski, Grazyna Kowalska, Aleksandra Szopa, Tomasz Baj, Henrique Douglas Melo Coutinho

**Affiliations:** 1 Pimenta Campus, Regional University of Cariri—URCA, Crato 63105-010, Brazil; 2 Brejo Santo Campus, Federal University of Cariri—UFCA, Brejo Santo 63260-000, Brazil; 3 Department of Mycology, Federal University of Pernambuco—University City, Recife 50670-901, Brazil; 4 Embrapa Tropical Agroindustry, Fortaleza 60511-110, Brazil; 5 Tauá Campus, State University of Ceara, Tauá 63660-000, Brazil; 6 São José Campus, CECAPE—College of Dentistry, Juazeiro do Norte 63024-015, Brazil; 7 Department of Analysis and Food Quality Assessment, University of Life Sciences in Lublin, 8 Skromna Str., 20-704 Lublin, Poland; 8 Department of Tourism and Recreation, University of Life Sciences in Lublin, 15 Akademicka Str., 20-950 Lublin, Poland; 9 Department of Clinical Pharmacy and Pharmaceutical Care, Medical University of Lublin, 1 Chodźki Str., 20-093 Lublin, Poland; 10 Department of Pharmacognosy with the Medicinal Plant Garden, Medical University of Lublin, 1 Chodźki Str., 20-093 Lublin, Poland

**Keywords:** popular medicine, umbu, flavonoids, fluconazole

## Abstract

This study aimed to identify the chemical composition of the *Spondias tuberosa* aqueous leaf and root extracts (EALST and EARST) and to evaluate their effect, comparatively, against opportunistic pathogenic fungi. Ultra-Performance Liquid Chromatography Coupled to a Quadrupole/Time of Flight System (UPLC-MS-ESI-QTOF) was employed for chemical analysis. *Candida albicans* and *C. tropicalis* standard strains and clinical isolates were used (CA INCQS 40006, CT INCQS 40042, CA URM 5974, and CT URM 4262). The 50% Inhibitory Concentration for the fungal population (IC_50_) was determined for both the intrinsic action of the extracts and the extract/fluconazole (FCZ) associations. The determination of the Minimum Fungicidal Concentration (MFC) and the verification of effects over fungal morphological transitions were performed by subculture in Petri dishes and humid chambers, respectively, both based on micro-dilution. UPLC-MS-ESI-QTOF analysis revealed the presence of phenolic and flavonoid compounds. The association of the extracts with fluconazole, resulted in IC_50_ values from 2.62 µg/mL to 308.96 µg/mL. The MFC of the extracts was ≥16,384 µg/mL for all tested strains, while fluconazole obtained an MFC of 8192 µg/mL against *C. albicans* strains. A reduction in MFC against CA URM 5974 (EALST: 2048 µg/mL and EARST: 1024 µg/mL) occurred in the extract/fluconazole association.

## 1. Introduction

Ethnobiology seeks to explain human knowledge of the environment and the way surrounding natural resources are manipulated [1]. The use of biological diversity by populations is one of the largest sources of new bioactive chemical molecule discoveries, among which medicinal plants stand out for their richness [2]. It is noteworthy that although the collection and use of medicinal plants is a common practice in different world cultures, this activity can pose challenges to conserving the used resources [3].

Thus, the local community using medicinal resources is the first to realize the reduction of its availability. For this reason, these individuals are the key holders of important knowledge which may determine species with priority for conservation, as well as for the production of strategies that allow their sustainable exploration [4].

The use of medicinal plants to cure diseases has been passed down from generation to generation. This knowledge contributes significantly to the propagation of therapeutic plant virtues that are commonly prescribed, despite the lack of an evaluation of their chemical constituents [5]. Thus, ethnobotanical, phytochemical, and pharmacological studies significantly collaborate to increase the knowledge surrounding natural products. This is especially due to the search for new therapeutic resources for the treatment of various diseases and to reduce dissatisfaction with conventional medicines [5,6].

*Spondias tuberosa* Arruda is an endemic Caatinga tree, popularly known as umbu. It serves as medicine and food for pollinators and domestic ruminants, in addition to producing fruits used *in natura* and processed to produce sweets, compote, pulps, and jellies. Despite its benefits, this plant faces many natural and anthropogenic threats that may lead to its extinction. Thus, scientific studies can add value and encourage its preservation [7,8].

In popular medicine, this species is used to treat infections, digestive disorders, and inflammatory conditions [9]. The leaves are used to treat diabetes, constipation, and stomach and uterine pain [10,11]. The stem bark is used for migraines, cholesterol control, and cicatrization [12,13]. The root tubers (infusion) are used to treat female discharge, a symptom possibly associated with *Candida* spp. infections [14].

The use of *S. tuberosa* (umbu) roots by the human population may cause damage in the future due to anthropic pressure on the species. The roots are vital to plant survival since they accumulate water and energy reserves. Thus, their exploitation could cause a decrease in plant populations in their environments [15]. With this in mind, and to add chemical and pharmacological information to the popular knowledge surrounding this species, the objective of this study was to comparatively identify and evaluate the chemical composition and antifungal action of the *S. tuberosa* aqueous leaf and root extracts compared to *C. albicans* and *C. tropicalis* standard strains and clinical isolates.

## 2. Results

The *S. tuberosa* Aqueous Leaf Extract (EALST) had a yield of 1.59%, and the *S. tuberosa* Aqueous Root Extract (EARST) had a yield of 0.78%. Preliminary phytochemical analysis of the extracts revealed the presence of several classes of metabolite (Table 1).

The High Definition Mass Spectrometry (UPLC–MS) chromatograms of the extracts are in the supplementary material (Supplementary Material—Figures S1 and S2). The results from the chromatographic analyses are shown in Table 2 and Table 3, where compound identification was performed based on their molecular ion mass, retention time, fragmentation pattern, and available data in the literature, shown in order of elution order, molecular formula, error, and major fragments (MS^2^).

The inhibition of 50% of the microorganismal population (IC_50_) for the EALST occurred at high concentrations for all tested strains. The EARST obtained an IC_50_ of 1306.6 µg/mL against CA URM 5974 and higher values for the other strains. Fluconazole (FCZ) presented an effect at concentrations ranging from 3.97 µg/mL to 88.08 µg/mL. In the extract/FCZ combination, a potentiation was observed for the EALST + FCZ combination against both *C. albicans* strains (11.80 and 2.62 µg/mL), and an antagonism was observed against *C. tropicalis* (177.41 and 308.96 µg/mL). For the EARST + FCZ combination, an antagonism was observed against CA URM 5974 (5.46 µg/mL), while a potentiation was observed against the other strains (ranging from 12.68 to 35.62 µg/mL). Despite the high concentrations, the results show the root extract was more effective than the leaf extract, which corroborate their use by the population (Table 4).

**Figure 1:**
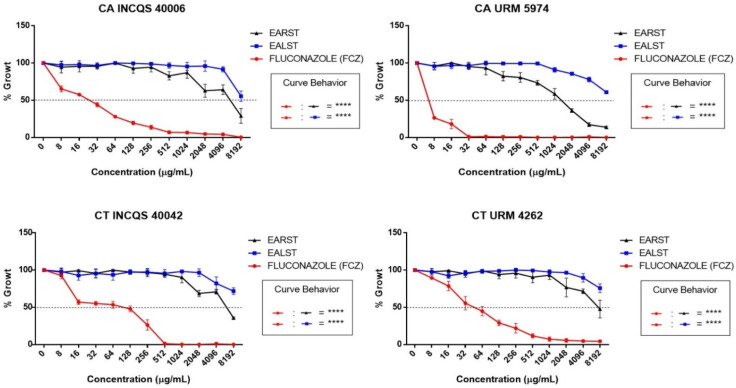
Cell viability curve of fluconazole and aqueous extracts of *Spondias tuberosa* against *Candida* strains. CA: *Candida albicans*; CT: *Candida tropicalis*; INCQS: National Institute of Quality Control in Health; URM: University Recife Mycology; EALST: Extract Aqueous of *Spondias tuberosa* Leaves; EARST: Extract Aqueous of *Spondias tuberosa* Root; FCZ: Fluconazole; ****—statistical significance with *p* < 0.0001.

In terms of an inhibitory action, as visualized by the cell viability curve, the extracts were not as effective as fluconazole (Figure 1). The MFC values for the extracts were ≥16,384 µg/mL against all tested strains, with a fungistatic effect being observed (Table 5).

**Figure 2:**
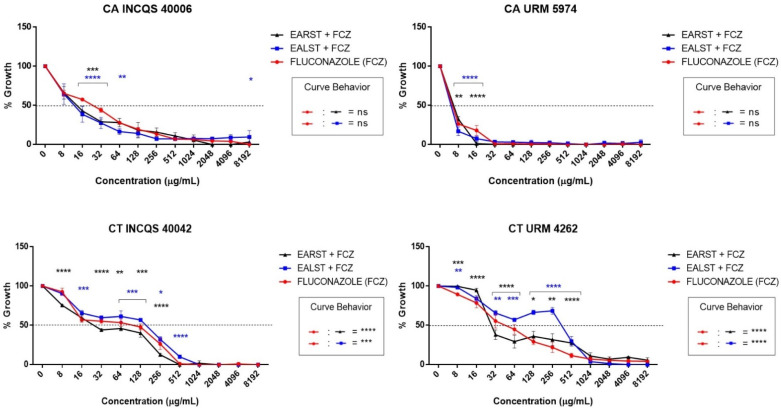
Evaluation of the modifying effect of fluconazole action by extracts. CA: *Candida albicans*; CT: *Candida tropicalis*; INCQS: National Institute of Quality Control in Health; URM: University Recife Mycology; EALST: Extract Aqueous of *Spondias tuberosa* Leaves; EARST: Extract Aqueous of *Spondias tuberosa* Root; FCZ: Fluconazole; ns: not significant; * *p* < 0.05; ** *p* < 0.01; *** *p* < 0.001; **** *p* < 0.0001—statistical significance.

In the extract/drug micro-dilution combinations, the EALST combination with fluconazole presented a potentiator effect against CA INCQS 40006 (16 µg/mL to 64 µg/mL), CA URM 5974 (8 µg/mL to 16 µg/mL), CT INCQS 40042 (16 µg/mL to 512 µg/mL), and CT URM 4262 (8 µg/mL at 512 µg/mL), with other concentrations obtaining results similar to fluconazole (Figure 2).

The EARST, when combined with fluconazole, presented as a potentiator against CA INCQS 40006 (16 µg/mL to 32 µg/mL), CA URM 5974 (8 µg/mL to 16 µg/mL), CT INCQS 40042 (8 µg/mL to 256 µg/mL) and CT URM 4262 (8 µg/mL to 512 µg/mL), obtaining a similar curve to that of the used drug for the remainder. In this essay, the leaf extract was more effective against the CA INCQS 40006 strain than the root extract. However, the root extract was more effective than the leaf extract against *C. tropicalis* strains.

**Figure 3:**
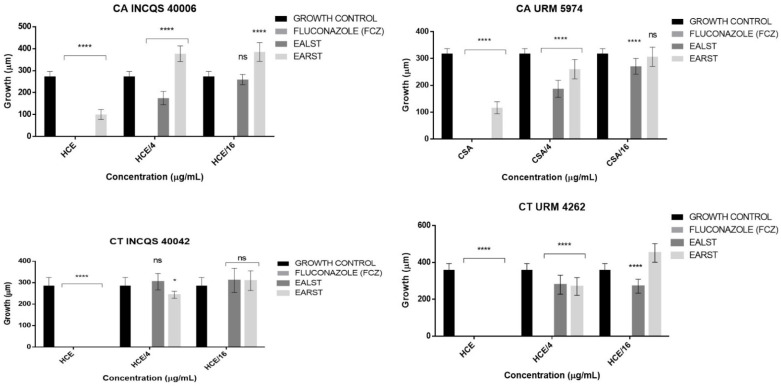
Effect of extracts and fluconazole on the morphological transition of *Candida* spp. CA: *Candida albicans*; CT: *Candida tropicalis*; INCQS: National Institute of Quality Control in Health; URM: University Recife Mycology; EALST: Extract Aqueous of *Spondias tuberosa* Leaves; EARST: Extract Aqueous of *Spondias tuberosa* Root; FCZ: Fluconazole; HCE: Higher Concentration Evaluated; ns: not significant; * *p* < 0.05; **** *p* < 0.0001—statistical significance.

As for the effect on yeast morphological transition, fluconazole inhibited the tested strains at all concentrations (Figure 3). The EALST inhibited all strains at the HCE concentration, with a 36.01% reduction in CA INCQS 40006 filament emission, 41.17% in CA URM 5974, and 22.35% in CT URM 4262 at the HCE/4 concentration, compared to the growth control. The results were not significant at the lowest concentration.

The EARST completely inhibited the morphological transition of the *C. tropicalis* standard and clinical isolate strains at the highest concentration (HCE), causing a 63.32% reduction in filamentation in CA INCQS 40006 and 63.36% in CA URM 5974. At the HCE/4 concentration, this reduction was 18.18% for CA URM 5974 and 25.11% for CT URM 4262, with the results being insignificant for the remaining strains. In the fungal morphology analysis, the leaf extract obtained greater effects than the root extract against the tested strains.

**Figure 4:**
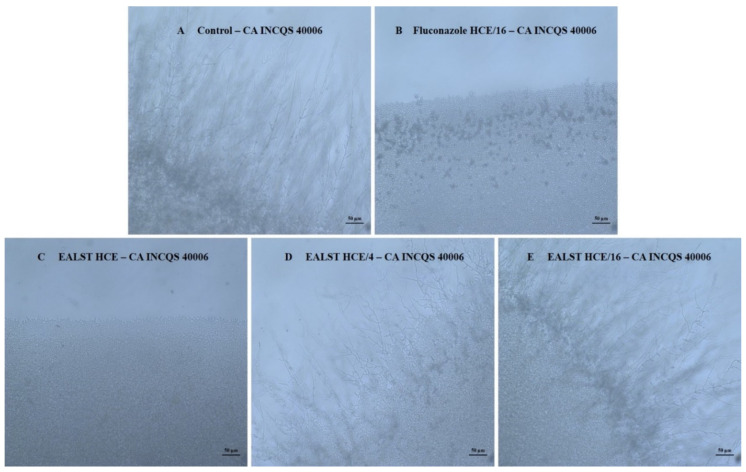
Demonstrative effect of EASTL and fluconazole on the morphological transition of *Candida albicans*. CA: *Candida albicans*; INCQS: National Institute of Quality Control in Health; EALST: Extract Aqueous of *Spondias tuberosa* leaves; FCZ: Fluconazole; HCE: Highest Concentration Evaluated. (**A**) Growth control, presence of filamentous structure; (**B**) Fluconazole control in HCE/16 concentration; (**C**) Effect of EALST on morphological transition in HCE concentration; (**D**) Effect of EALST on morphological transition in HCE/4 concentration; (**E**) Effect of EALST on morphological transition in HCE/16 concentration.

Figure 4A shows that in the CA INCQS 40006 growth control, filament formation with extensive hyphae is evident. Figure 4B shows the effect of fluconazole at the lowest concentration (HCE/16), where a total filament reduction can be observed. The progressive effect of the EALST on *C. albicans* pleomorphism inhibition can be seen in Figure 4C–E.

## 3. Discussion

Qualitative phytochemical studies previously performed with the crude ethanolic leaf and bark extracts from *Spondias* sp. and *S. tuberosa* revealed the presence of flavonoids, triterpenes, and alkaloids, these classes being found in both extracts in the present study. In addition, steroids, tannins, terpenoids, saponins, coumarins, monoterpenes, sesquiterpenes, and diterpene compounds were also detected [22,23]. The authors of [9] also revealed the presence of phenolic compounds and flavonoids in the *S. tuberosa* leaf hydroethanolic extract using the thin-layer chromatography technique.

In the preliminary phytochemical analysis of the hydroalcoholic extracts of leaves and roots of *S. tuberosa* carried out by [24], different results were observed than those found in this study. This may be related to the type of solvent and polarity [25] used, because the aqueous extract transports substances of a polar nature while the hydroalcoholic extract transports polar and non-polar substances.

In a previous study by [24], no alkaloids were detected in the hydroalcoholic extract of the root, while in the present study, they were found in the aqueous extract of the same organ. Although alkaloids are most effectively extracted with non-polar and medium-polar extracts [26], they can also be extracted using water as a solvent, which, when heated, can become capable of extracting ostensibly hydrophobic compounds [27], justifying the presence of alkaloids in the aqueous extract by infusion. Unlike the hydroalcoholic extract that revealed the presence of steroids and flavonoids of the flavone, flavonol, and xanthone type, in the aqueous extract, their presence was not evidenced. According to [26], steroids can be extracted by ethanol, explaining the fact that they were detected in a water/alcohol solvent mixture. The absence of flavonoids of the flavone, flavonol, and xanthone types in the aqueous extract is due to the fact that they are more efficiently extracted by the ethanol solvent compared to water [26,28]. Flavonoids, such as anthocyanin, anthocyanidin, leucoanthocyanidins, catechins, and triterpenoids, were present only in the aqueous extract of the leaves, showing that, although both solvents were able to extract the compounds [26,28], the water alone was more effective than the hydroalcoholic mixture in the extraction.

In the study by [24], the authors analyzed the chemical composition of the *S. tuberosa* leaf and root hydroalcoholic extracts by UPLC-MS-ESI-QTOF. They reported the presence of dehydrophasic hexose acid, caffeine-D-glucose, (±)-naringenin, anacardic acid, kaempferol-7-Oglucuronide, 2-hydroxy-3,4-dimethoxybenzoic acid, quercetin O-pentoside, ramnetin, and monogaloyl glucose.

The aqueous *Spondias mangifera* Willd. root extract was investigated for its phytochemistry and revealed the presence of flavonoids, saponins, tannins and phenolic compounds [29]. The study by [30] identified the presence of alkaloids and flavonoids similar to those found in the present study, in addition to detecting tannins, steroids, and saponins in the crude *S. mombin* leaf and fruit extracts.

The authors of [31] identified the compounds kaempferol and monogalloyl-glucose, also found in the present analysis, in the LC-MS/MS analysis of the *Mangifera indica* L. juice, a species belonging to the same family as *S. tuberosa*. In a study using the flavonoid kaempferol, a compound identified in the leaf extract, its minimum inhibitory concentration (MIC) was determined using the broth micro-dilution technique against different species from the *Candida* genus. The MIC against *C. parapsilosis* ranged from 32–128 µg/mL^−1^, 32–64 µg/mL^−1^ for *C. metapsilosis*, and 64 µg/mL^−1^ for *C. orthopsilosis* and *C. krusei*, respectively [18]. The authors of [32] obtained an MIC_90_ ranging from 256 µg/mL to 512 µg/mL in a susceptibility test against different *C. albicans* strains using kaempferol, where fluconazole ranged from 0.5 µg/mL to 2048 µg/mL.

In popular medicine, *S. tuberosa* leaves are used for uterine pain [11], and the root tubers (infusion) are used to treat female discharge, symptoms that may be associated with *Candida* spp. Infections [14]. Based on these ethnobotany studies, it is assumed that the *S. tuberosa* therapeutic form against fungi is its use in sitz baths and tea administration, where the ingestion of the natural product may reduce an infection caused by the microorganism.

The work by [33] evaluated the antifungal activity of hexane extract of *S. tuberosa* leaves against standard strains (*Cryptococcus gattii* ATCC 24065 and *Cryptococcus neoformans* ATCC 24067) and clinical isolates (*C. gattii* 547 and *C. neoformans* RN01), using the technique of micro-dilution to determine minimum inhibitory concentration (MIC), the extract inhibited the growth of all tested strains of *C. neoformans* and *C. gattii* with MICs ranging from 0.78 to 3.12 mg/mL. In the work by [34], using the same extract and technique mentioned above, as well as determining the minimum fungicide concentration (CFM) with strains of *C. albicans* URM 5901, *C. parapsilosis* URM 6951, *C. glabrata* URM 4246, and *C. krusei* URM 6391, the tests revealed that the hexane extract of leaves of *S. tuberosa* was able to inhibit the growth of *C. albicans* and *C. glabrata* with MIC_50_ values from 2 to 0.078 mg/mL, respectively, and no fungicidal effect was detected.

The study by [24] observed similar results to those of the aqueous *S. tuberosa* extracts when evaluating the *S. tuberosa* hydroalcoholic leaf and root extracts using the same techniques and strains in the present study. The results corroborate with the present results, showing the *S. tuberosa* extract presented no fungicidal effect against *Candida* strains at different extractions.

The authors of [35] carried out a study with the *S. tuberosa* bark ethanolic extract against different species from the *Candida* genus using the disk diffusion technique and found the extract was unable to inhibit the growth of any tested species.

Species from the same genus as *S. tuberosa* have been investigated for their antifungal activity. The aqueous fraction from the *Spondias pinnata* (L. f.) Kurz. fruit exocarp was ineffective against *C. albicans* and *Saccharomyces cerevisiae* yeasts using the disk-diffusion method [36]. The authors of [37] obtained an inhibition of 10 mm and 4 mm, respectively, against *C. albicans* with the chloroform and dichloromethane extracts from *Spondias dulcis* leaves using the same technique. A study showed a minimum inhibitory concentration (MIC) of 0.0156 mg, 0.2500 mg, and 0.2500 mg, respectively, against *C. albicans* using the *Spondias mombin* L. leaf, bark, and root aqueous extracts [38].

Methanolic extract and n-hexane from the leaves and bark of *S. mombin* were tested using the agar diffusion method, revealing that the plant extracts did not act against *Aspergillus niger* and *C. albicans* [39]. In the study by [40], with aqueous extract (leaves) and hydroethanolic extract (bark) of *S. mombin* using the same techniques as the present study and the standard species of *C. albicans* INCQS 40006, *C. krusei* INCQS 40095, and *C. tropicalis* INCQS 40042, it was observed there was no fungicidal effect and that the plant extracts had effects at higher concentrations, results similar to those of the present research, despite being a plant of different species and extract.

Some of the mechanisms attributed to fungal pathogenicity are associated with dimorphism, the ability of yeast to grow hyphae or pseudohyphae. Other characteristics, in addition, contribute to intensifying the colonization and infectious ability of fungal species [41,42,43].

In a study by [44], flavonoids present in extracts were capable of forming complexes with soluble proteins present in the fungal cell walls and being capable of rupturing the fungal membrane due to their lipophilic nature [45]. They were also able to inhibit the budding process and decrease Ca^2+^ and H^+^ homeostasis [46]. Thus, the fungal morphology action observed in the present study was probably due to the presence of these compounds in the extracts. The difference in results obtained for the different *Candida* species tested may be associated with variations in pathogenicity and virulence between species [47,48].

The authors of [49] found that flavonoids may be responsible for the reduction in hyphal growth, with the compound kaempferol, which was also detected in the leaves of the present study, being identified in its honey flavonoid extract.

Yeast virulence inhibition by *S. tuberosa* extracts may be associated with its metabolites acting synergistically. However, further studies are required to ascertain this since other compounds present in the extracts, in addition to flavonoids, may also be involved in the process.

## 4. Materials and Methods

### 4.1. Botanical Material Collection and Identification

The *Spondias tuberosa* Arruda botanical material was obtained under permission from the Biodiversity Authorization and Information System—SISBIO under number 64293-1, from the community of Lameiro (07°15′03.1″ South latitude and 39°23′48″ longitude West of Greenwich), in the municipality of Crato, southern Ceará, Brazil. Leaves and roots were harvested from seven individual plants between 8:00 a.m. and 9:30 a.m. in June 2018. An exsiccate was produced from the collected material. A specimen was deposited in the Herbarium Dárdano de Andrade Lima (HCDAL) from the Regional University of Cariri—URCA with the herbarium number 13728, identified by Professor Ma. Ana Cleide Alcantara Morais Mendonça.

### 4.2. Extract Acquisition and Chemical Analysis

Young leaves and roots, which were cut into smaller fragments to increase their contact surface with the extractor (water), were used for preparing the aqueous extracts by infusion. The infusion was prepared by pouring boiling water on the leaves at a rate of 133.2 g for every two liters of water, with the container being closed and left to rest for 15 min after cooling [50] before subsequent filtration.

The extracts were dried by spray drying with a Mini Spray Dryer MSDi 1.0 (Labmaq do Brasil), using a 1.2 mm spray nozzle, under the following operating conditions: (a) flow control: 500 mL/h; (b) inlet temperature: 130 ± 2 °C; (c) outlet temperature: 74 ± 2 °C; (d) atomization air flow: 45 L/min; (e) blower flow rate: 1.95 m^3^/min [51].

The extracts were submitted to preliminary phytochemical analysis based on qualitative methods proposed by [52]. In these experiments, characterization of the main classes of special metabolites was performed via chemical reactions that resulted in the development of color and/or precipitates, after the addition of specific reagents, that are characteristic for the following classes of metabolites: alkaloids, steroids, phenols, flavonoids, tannins, and triterpenoids. Solutions were prepared by dissolving 1.0 g of each crude extract with 100 mL of distilled water. Subsequently, 3 mL aliquots from these solutions were added to test tubes to characterize the groupings. All experiments were performed in triplicate, and the metabolite classes were identified as present (+) or absent (−).

The identification of compounds present in the extracts was performed in an Acquity^®^ UPLC system coupled to a Quadrupole/Time of Flight Time (QTOF) system (Waters Corporation, Milford, CT, USA), at the Chemical and Natural Products Laboratory, Embrapa Tropical Agroindustry (Fortaleza, Ceará, Brazil). Chromatographic runs were performed on a Waters Acquity^®^ UPLC BEH column (150 × 2.1 mm; 1.7 µm), with a fixed temperature of 40 °C, mobile phases with 0.1% formic acid (A) and acetonitrile with 0.1% formic acid (B), gradient ranging from 2% to 95% B (15 min), at a flow rate of 0.4 mL/min and injection volume of 5 µL. The ESI^+^ mode was acquired in the 110–1180 Da range, 120 °C fixed source temperature, 350 °C desolvation temperature, 500 L/h desolvation gas flow, and 3.2 kV capillary voltage. The ESI^-^ mode was obtained in the 110–1180 Da range, 120 °C fixed source temperature, 350 °C desolvation temperature, 500 L/hr desolvation gas flow, 0.5 V extraction cone, and 2.6 kV capillary voltage. Leucine encephalin was used as the lock mass. The acquisition mode was MSE (high energy mass spectrometry) [53]. The instrument was controlled by Masslynx^®^ 4.1 software (Waters Corporation, Milford, CT, USA).

### 4.3. Antifungal Assays

#### 4.3.1. Microorganisms

For the antifungal activity evaluation, two Candida albicans, CA INCQS 40006 and Candida tropicalis, CT INCQS 40042 standard fungal strains, were obtained from the Oswaldo Cruz Culture Collection of the National Institute for Quality Control in Health (INCQS). The two Candida albicans, CA URM 5974 and Candida tropicalis, CT URM 4262 clinical isolates, were provided by the Recife Micoteca University (URM) of the Federal University of Pernambuco—UFPE.

#### 4.3.2. Culture Media

The Sabouraud Dextrose Agar (SDA, KASVI, Laboratorios Conda S.A., Madrid, Spain) solid medium, prepared according to the manufacturer’s instructions, and the double concentrated Sabouraud Dextrose Broth (SDB, HIMEDIA, Mumbai, India) liquid medium, were used for the antifungal activity evaluations. The Potato Dextrose Agar (PDA, Difco, Becton, Dickinson and Company, Franklin Lakes, NJ, USA) depleted by dilution and supplemented with agar was used for the fungal morphology analysis. The media were solubilized with distilled water and autoclaved at 121 °C for 15 min.

#### 4.3.3. Inoculum Preparation

All strains were replicated and initially kept in test tubes containing inclined SDA, refrigerated at 8 °C, at the Cariri—LMAC Applied Mycology Laboratory of the Regional University of Cariri—URCA. For the fungal inoculum preparation, these were first cultured in an SDA medium poured into Petri dishes at 37 °C for 24 h (overnight). From these, microorganism suspensions were prepared in tubes containing 4 mL of a sterile solution (0.9% NaCl). These suspensions were then shaken with the aid of a vortex apparatus, and their turbidity was compared and adjusted to that shown by the McFarland scale 0.5 barium sulfate suspension, which corresponds to an inoculum of approximately 10^5^ colony-forming units/mL—CFU/mL [54]. The inocula were used for intrinsic and drug-combined antifungal activity tests performed by broth micro-dilution and Minimum Fungicide Concentration (MFC).

#### 4.3.4. Used Drugs and Reagents

Dimethylsulfoxide (DMSO, Merck, Darmstadt, Germany) was used for extract dilution. The antifungal fluconazole 150 mg (prati—donaduzzi and CIA LTDA, Toledo, Brazil) was diluted in water and used as a reference drug for the tests since it is the most commonly used antifungal in the Brazilian healthcare system. To prepare the initial extract solution, 0.15 g was weighed and solubilized in 1 mL of DMSO. Subsequently, the extracts and fluconazole were further diluted in sterile distilled water to obtain the desired initial concentration for the tests (16,384 µg/mL).

#### 4.3.5. IC_50_ Determination and Cell Viability Curve

This experiment was performed using the broth micro-dilution technique with 96 well plates. Each well was filled with 100 μL of SDB containing 10% fungal inoculum, followed by 100 μL of the natural product or fluconazole at the same concentrations, followed by serial micro-dilution up to the penultimate well starting from a concentration of 8192 to 8 µg/mL. The last well was reserved for growth control [55], with changes in concentration. Dilution controls for the natural product and fluconazole (with 0.9% sodium chloride solution replacing the fungal inoculum), as well as media sterility controls, were also performed. All tests were performed in quadruplicate. The plates were incubated at 37 °C for 24 h and then read on an ELISA spectrophotometer (Kasuaki—DR—200Bs—BI, Stockholm, Sweden) at a wavelength of 450 nm. The results obtained from the ELISA readings were used to construct the cell viability curve and determine the IC_50_ of the extracts and fluconazole [56].

#### 4.3.6. Determination of Minimum Fungicidal Concentration (MFC)

A sterile rod was placed into each well of the plate used in the micro-dilution test, following 24 h of incubation and the ELISA reading (except for the sterility control), to perform this test. After homogenizing the solution contained in the well, subcultures were performed in a Petri dish containing SDA and a guide card by transferring a small aliquot from the test solution (medium + inoculum + natural product). The plates were incubated at 37 °C for 24 h and checked for growth or non-growth of Candida colonies [57], with modifications. The MFC was defined as the lowest concentration capable of inhibiting fungal colony growth.

#### 4.3.7. Evaluation of the Modifying Effect on Fluconazole Action 

The effect of combining the extracts with the reference drug (fluconazole) was performed to verify the potentiating action of the antifungal by adding the extracts. The solutions containing the extracts were tested at sub-inhibitory concentrations (MFC/16) according to the methodology used by [58], with changes in concentrations. When natural extracts increase the action of the antifungal, this is considered a potentiating effect; when it impairs the activity of the drug, it is considered an antagonistic effect. The plates were filled with 100 µL of the medium + inoculum + extract, being then micro-diluted with 100 µL of fluconazole; this was mixed into the first well by serial micro-dilution at a 1:1 ratio up to the penultimate well. Medium sterility controls were also performed. All tests were performed in quadruplicate. The plates were incubated at 37 °C for 24 h. The readings were performed in an ELISA spectrophotometry device (Kasuaki—DR—200Bs—BI, Stockholm, Sweden) [56].

#### 4.3.8. Effect of the Extracts and Fluconazole on the Micromorphology of *Candida* spp.

Humid chambers were prepared with sterile microscopy slides for yeast observation to analyze changes in fungal morphology by the extract and fluconazole via the inhibition or reduction of hyphae emission. Three mL of depleted PDA medium with HCE—8192 µg/mL, HCE/4—2048 µg/mL, and HCE/16—512 µg/mL (Highest Concentration Evaluated) natural product and fluconazole concentrations were added to the chambers. Aliquots from the subcultures were taken to make two parallel striations in the solid medium (PDA), which were subsequently covered with a sterile cover slip. The chambers were incubated for 24 h (37 °C), inspected and recorded by an optical microscope (AXIO IMAGER M2-3525001980-ZEISS-Germany) using a 20× objective. A control for yeast growth (nutrient restriction-stimulated hyphae and pseudohyphae) was performed, as well as a control with the conventional antifungal fluconazole for comparative purposes. The tests were performed according to [59,60] with modifications. The Zen 2.0 software was used to measure hyphae and pseudohyphae extensions, where images were taken from each complete striae, and five random images from these were used for statistical analysis [61].

### 4.4. Statistical Analysis

A two-way ANOVA was applied to each sample, comparing the values for each extract concentration using Bonferroni’s post-hoc test, in which *p* < 0.05 and *p* < 0.0001 are considered significant and *p* > 0.05 is insignificant. IC_50_ values were obtained by nonlinear regression with unknown interpolation of standard curves obtained from fungal growth values as a function of the extract concentration, expressed in μg/mL. The GraphPad Prism software, version 6.0, was used for statistical analysis.

The measurements of the entire striae edge and the regions where hyphae growth occurred were performed for virulence analysis. Subsequently, all identified hyphal filaments were measured from five randomly selected regions of each striae and concentration. Hyphal filament length was averaged and analyzed by an ANOVA, followed by Bonferroni’s correction for multiple comparisons according to the product concentration.

## 5. Conclusions

The *S. tuberosa* aqueous leaf and root extracts presented in their composition mainly phenolic and flavonoid compounds, which may have contributed to the isolated and synergistic activity observed in the tests. In this study, the leaves and roots had their bioactive potential scientifically evaluated by tests, where the extracts, together with fluconazole, were able to reduce its concentration, increasing the effectiveness of the drug. The EALST was shown to be more effective than the EARST in fungal morphology inhibition, an important mechanism of *Candida* virulence, which may be important from a plant conservation point of view since the leaves may be a less harmful usage option and may lower the impact caused by using the species’ roots. However, further in-depth studies are needed to identify the active compounds and prove their applicability, safety, mechanism of action, and the genes involved.

## Figures and Tables

**Table 1:** Preliminary phytochemical analysis of aqueous extracts of leaves and roots of *Spondias tuberosa*.

	Special Metabolite Classes (SMC)									
	SMC1	SMC2	SMC3	SMC4	SMC5	SMC6	SMC7	SMC8	SMC9	SMC 10
EALST	+	-	-	+	+	+	-	-	+	+
EARST	+	-	-	+	-	+	-	-	+	+

SMC1: Phenols; SMC2: Tannins; SMC3: Flavonoids of the flavone, flavonol, and xanthone type; SMC4: Flavonoids of the anthocyanin and anthocyanidin type; SMC5: Flavanoid of leucoanthocyanidins, catechins; SMC6: Flavanone type flavanoid; SMC7: Flavanoid type flavanoid; SMC8: Steroids; SMC9: Triterpenoids; SMC10: Alkaloids; (+): positive; (-): missing.

**Table 2:** UPLC-MS-ESI-QTOF identification of compounds from aqueous leaves extract of *Spondias tuberora* (Positive Mode).

Peak no.	Rt min	[M + H]^+^ Observed	Product Ions (MS/MS)	Molecular Formula	Ppm Error	Putative Name	Ref.
1	2.52	214	198	C_4_H_6_S_5_	4.6	No identified	
2	2.67	185	-	C_8_H_8_O_5_	1.5	Methyl digallate	[16]
3	2.74	445	283	C_21_H_32_O_10_	2.5	Dihydrophaseic acid hexose	[17]
4	4.19	449	303	C_21_H_20_O_11_	0.4	Quercetin rhamnoside	[17]
5	4.49	167	120	C_9_H_12_NO_2_	−4.2	No identified	-
6	4.35	449	287	C_21_H_20_O_11_	0.2	Kaempferol hexoside	[18]
7	4.55	463	287	C_21_H_18_O_12_	0.4	Kaempferol glucuronide	[18]
8	4.62	477	287	C_22_H_20_O_12_	−4.4	Kaempferol-methyl-glucuronide	[18]
9	4.80	287	-	C_15_H_10_O_6_	−2.5	Kaempferol	[18]
10	5.49	681	303	C_34_H_48_O_14_	−1.6	No identified	-

**Table 3:** UPLC-MS-ESI-QTOF identification of compounds from aqueous roots extract of *Spondias tuberora* (Negative Mode).

Peak no.	Rt min	[M − H]^−^ Observed	Product Ions (MS/MS)	Molecular Formula	Ppm Error	Putative Name	Ref.
1	2.89	271	-	C_15_H_12_O_5_	3.8	No identified	-
2	3.16	377	237, 189	C_25_H_14_O_4_	2.1	No identified	-
3	3.20	331	169	C_9_H_16_O_13_	2.8	Monogalloyl-glucose	[17]
4	3.51	197	-	C_9_H_10_O_5_	1.8	Hydroxy-dimethoxybenzoic acid	[16]
5	4.55	461	285	C_22_H_22_O_11_	2.4	Kaempferol-glucuronide	[17]
6	4.66	433	301	C_20_H_18_O_11_	3.0	Quercetin-pentoside	[19,20]
7	4.89	339	-	C_22_H_28_O_3_	−3.4	No identified	-
8	5.07	361	317	C_20_H_26_O_6_	2.1	Giberellin GA19	[17]
9	5.37	537	479, 375	C_30_H_18_O_10_	4.5	Agathisflavone	[21]
10	5.55	315	-	C_16_H_12_O_7_	4.1	Rhamnetin	[19,20]
11	5.92	341	297	C_22_H_30_O_3_	4.4	Anacardic acid 1	[17]
12	7.00	343	299	C_22_H_32_O_3_	−4.7	Anacardic acid 2	[17]
13	7.59	345	301	C_22_H_34_O_3_	−4.1	Anacardic acid 3	[17]

**Table 4:** 50% Inhibitory Concentrations (IC_50_) of microorganisms (µg/mL) by aqueous extracts of *Spondias tuberosa*.

	CA INCQS 40006	CA URM 5974	CT INCQS 40042	CT URM 4262
EALST	8305.3	11,090.9	16,288.03	13,335.7
EARST	5344.8	1306.6	6678.9	7785.9
FLUCONAZOLE	22.74	3.97	88.08	47.30
EALST + FCZ	11.80	2.62	177.41	308.96
EARST + FCZ	12.68	5.46	30.42	35.62

CA: *Candida albicans*; CT: *Candida tropicalis*; INCQS: National Institute of Quality Control in Health; URM: University Recife Mycology; EALST: Aqueous Extract of *Spondias tuberosa* Leaves; EARST: Aqueous Extract of *Spondias tuberosa* Root; FCZ: Fluconazole.

**Table 5:** Minimum Fungicidal Concentration (MFC) of microorganisms (µg/mL) by extracts, fluconazole, and their combination.

	CA INCQS 40006	CA URM 5974	CT INCQS 40042	CT URM 4262
EALST	≥16,384	≥16,384	≥16,384	≥16,384
EARST	≥16,384	≥16,384	≥16,384	≥16,384
FLUCONAZOLE	8192	8192	≥16,384	≥16,384
EALST + FCZ	8192	2048	≥16,384	8192
EARST + FCZ	≥16,384	1024	≥16,384	8192

CA: *Candida albicans*; CT: *Candida tropicalis*; INCQS: National Institute of Quality Control in Health; URM: University Recife Mycology; EALST: Aqueous Extract of *Spondias tuberosa* Leaves; EARST: Aqueous Extract of *Spondias tuberosa* Root; FCZ: Fluconazole.

## Data Availability

Data are contained within the article.

## Supplementary Materials

The following supporting information can be downloaded at: https://www.mdpi.com/article/10.3390/molecules28010305/s1, Figure S1. High Definition Mass Spectrometry (UPLC–MS) Chromatogram of the Aqueous Extract of Leaves from Spondias tuberosa in the Negative Ionic Mode; Figure S2. High Definition Mass Spectrometry (UPLC–MS) Chromatogram of the Aqueous Extract of Roots from Spondias tuberosa in the Negative Ionic Mode.

## Sample Availability

Samples of the compounds are available from the authors after request.
