# Supplementary material for: UPLC-MS-ESI-QTOF Analysis and Antifungal Activity of Aqueous Extracts of Spondias tuberosa
Source: Molecules. 2022 Dec 30;28(1):305. doi: 10.3390/molecules28010305 (PMC9822456; doi:10.3390/molecules28010305)
Supplement: Supplementary file 1 [file molecules-28-00305-s001.zip › molecules-2046663-supplementary.pdf]

Supplementary material

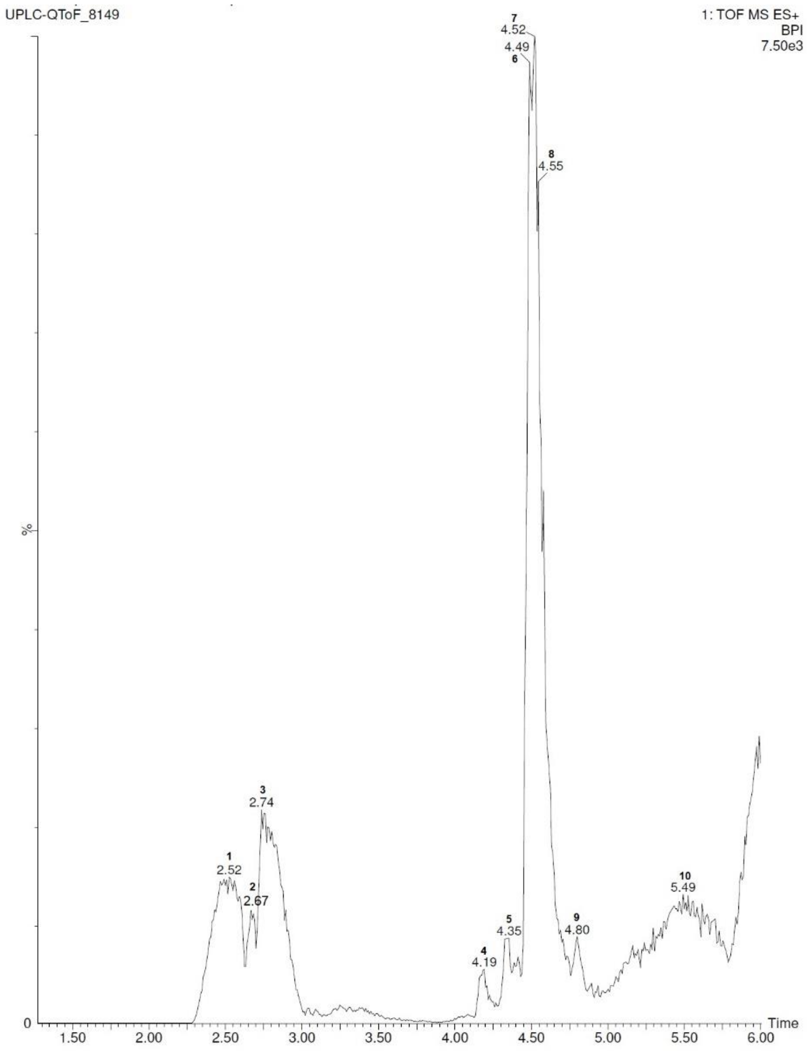

Figure S1 - High Definition Mass Spectrometry (UPLC–MS) Chromatogram of the Aqueous Extract of Leaves from *Spondias tuberosa* in the Positive Ionic Mode.

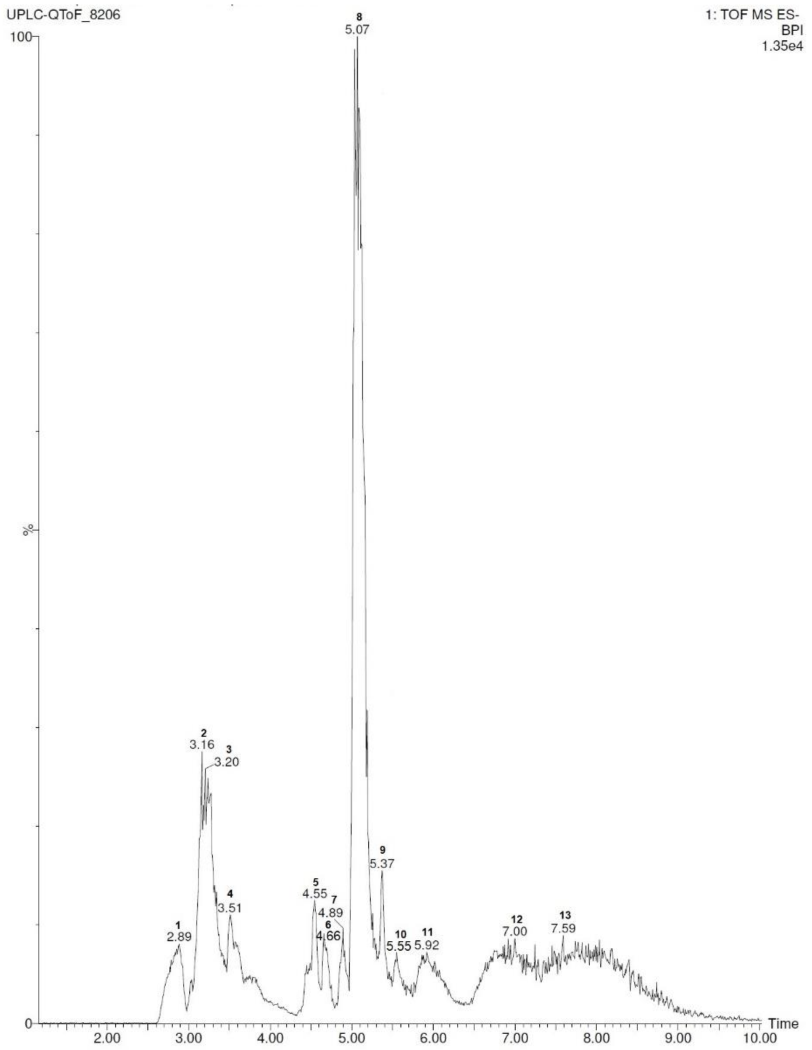

Figure S2 - High Definition Mass Spectrometry (UPLC–MS) Chromatogram of the Aqueous Extract of Roots from *Spondias tuberosa* in the Negative Ionic Mode.

.
